# DNA Methylation Fine-Tunes Light- and Hormone-Responsive Growth Plasticity in *Arabidopsis* Seedlings

**DOI:** 10.3390/ijms27021034

**Published:** 2026-01-20

**Authors:** Emanuela Talarico, Eleonora Greco, Adriana Chiappetta, Fabrizio Araniti, Leonardo Bruno

**Affiliations:** 1 Unit of Plant Biology, Department of Biology, Ecology and Earth Sciences (DiBEST), University of Calabria, Arcavacata of Rende, 87036 Cosenza, Italy; emanuela.talarico@unical.it (E.T.); eleonora.greco@unical.it (E.G.); adriana.chiappetta@unical.it (A.C.); 2 Department of Agricultural and Environmental Sciences-Production, Landscape, Agroenergy (Di.S.A.A.), University of Milano, 20133 Milano, Italy; fabrizio.araniti@unimi.it

**Keywords:** DNA methylation, *Arabidopsis thaliana*, photomorphogenesis, auxin, gibberellin, epigenetic regulation, growth plasticity, circadian clock, seedling development

## Abstract

DNA methylation regulates plant growth by modulating gene expression; however, its contribution to hormone responsiveness and photomorphogenesis remains only partially understood. We examined *Arabidopsis thaliana* DNA methylation mutants *met1* and *drm1*, *drm2*, and *cmt3* (*ddc*) under defined light regimes and following exogenous treatments with auxin, gibberellin, and the auxin transport inhibitor TIBA. Hypocotyl elongation and cotyledon expansion exhibited strong light dependency across all genotypes, with *met1* seedlings developing a consistently reduced cotyledon area and *ddc* seedlings displaying impaired hypocotyl elongation under specific light qualities. Exogenous auxin inhibited growth in all genotypes, whereas GA_3_ promoted elongation in hypocotyls and roots (by approximately 75–80% and 15–35%, respectively, in Col0 and *met1*), with *ddc* exhibiting delayed and non-linear dose-dependent sensitivity. Quantitative RT–PCR analysis revealed differential expression of genes involved in auxin transport (*PIN1*, *PIN3*, *PIN7*), auxin signalling (*ARF7*, *IAA3*, *LAX3*), circadian regulation (*TOC1*, *LHY*, *CCA1*), and light signalling (*PIFs*, *HY5*, *HYH*), supporting a link between DNA methylation status and coordinated regulation of hormone-, light-, and clock-controlled transcriptional networks. Together, these findings demonstrate that MET1- and DRM/CMT-dependent methylation pathways integrate epigenetic regulation with environmental and hormonal cues, modulating the intensity, timing, and organ specificity of growth responses, thereby fine-tuning growth plasticity during early *Arabidopsis* seedling development.

## 1. Introduction

Epigenetic regulation allows both plants and animals to modulate gene expression in response to developmental and environmental cues. Such mechanisms provide organisms with phenotypic flexibility, which is essential for growth, adaptation, and long-term genomic stability [1]. DNA methylation is one of the most widely studied epigenetic marks and plays a central role in transcriptional regulation, chromatin organisation, and genome integrity. By influencing gene accessibility without altering DNA sequence, DNA methylation enables organisms to fine-tune developmental programmes in response to fluctuating external conditions. Although methylation occurs in both plants and animals, its genomic patterns, enzymatic machinery, and biological functions differ substantially between kingdoms [2,3].

In plants, early seedling development represents a particularly dynamic phase in which epigenetic regulation interacts tightly with environmental signals [4,5]. Light acts as a major developmental cue, directing the transition between skotomorphogenesis, characterised by elongated hypocotyls and closed cotyledons in darkness, and photomorphogenesis, which promotes hypocotyl growth inhibition, cotyledon expansion, and chloroplast differentiation [6]. This developmental switch is tightly coordinated with hormonal signalling pathways, particularly auxin and gibberellins, which act downstream of light perception to regulate cell elongation and organ growth [7].

Because this switch requires precise transcriptional reprogramming, it provides an ideal context for investigating how defects in DNA methylation influence the integration of hormonal and environmental signals. Despite these shared principles, the organisation and function of DNA methylation differ markedly between plants and animals.

In mammals, DNA methylation is largely restricted to CpG dinucleotides and undergoes extensive reprogramming during reproduction. Gametes are demethylated early in development, followed by widespread de novo methylation after implantation. CpG methylation is catalysed by the DNA methyltransferases DNMT1, DNMT3A, and DNMT3B, which maintain or establish methylation patterns genome-wide [1]. In contrast, plants methylate cytosines in three sequence contexts: CG, CHG, and CHH (where H can be A, T, or C). They also employ a diversified set of methyltransferases, allowing both maintenance and de novo methylation [8,9]. This multilayered methylation system provides plants with increased regulatory complexity and flexibility, particularly in response to environmental stimuli.

The maintenance of CG methylation in *Arabidopsis thaliana* L. Heynh is mediated by MET1, which is functionally analogous to mammalian DNMT1 [2]. Different *met1* alleles display variable phenotypes: *met1-1* shows late flowering and reduced fertility, whereas *met1-2* presents milder developmental defects [10,11,12]. Non-CG methylation is maintained by chromomethyltransferases (CMTs), and de novo methylation is catalysed primarily by DOMAINS REARRANGED METHYLTRANSFERASES DRM1 and DRM2 through the RNA-directed DNA methylation (RdDM) pathway [3]. DRM2 is the principal active enzyme, whereas DRM1 provides partial redundancy at lower expression levels. CMT3 maintains CHG methylation and contributes to heterochromatic CHH methylation, and combined loss of *DRM1*, *DRM2*, and *CMT3* (*ddc* mutants) produces pleiotropic developmental defects including reduced stature, curled leaves, and partial sterility [13,14,15,16].

However, the specific contribution of CG versus non-CG methylation to hormone-mediated seedling growth remains incompletely resolved.

DNA methylation interacts closely with plant hormonal signalling to regulate growth and development. Auxin (indole-3-acetic acid, IAA) is central to cell elongation, division, and meristem patterning [17,18]. Auxin gradients, established and maintained by polar transporters such as PIN1, PIN3, PIN7, and influx carriers like LAX3, are critical for organ patterning and growth. Auxin responses are mediated by Auxin Response Factors (ARFs), which are regulated through the TIR1/AFB receptor-mediated degradation of Aux/IAA repressors [18,19]. Disruption of DNA methylation can affect both auxin transport and perception, leading to altered sensitivity and organ-specific growth defects, as reported in *ddc* mutants [20,21,22]. Nevertheless, how methylation-dependent regulation modulates auxin responsiveness across different seedling organs and environmental contexts remains largely unexplored.

Gibberellins (GAs) represent another key hormonal pathway interacting with auxin and light signalling to control cell elongation and organ growth. GAs promote growth via the degradation of DELLA proteins through the GID1 receptor and SCF/SLY1 ubiquitin ligase, relieving repression of PIFs, BZR1, and ARF6 to coordinate elongation [19]. Crosstalk between GA and auxin is crucial for hypocotyl elongation, root growth, and cotyledon expansion. Epigenetic defects in DNA methylation, particularly in non-CG contexts, appear to modulate GA responses in a genotype- and organ-specific manner [20,23]. However, direct comparative analyses of GA responsiveness in CG versus non-CG methylation mutants during early seedling development are still limited.

Light perception is also tightly linked to both hormone signalling and DNA methylation. Phytochrome-interacting factors (PIFs) and light signalling components such as HY5, HYH, and EID1 integrate environmental cues with growth programmes. In methylation mutants, these regulators are often misexpressed: PIF3 is downregulated in *met1* and *ddc*, whereas PIF4, PIF5, and PIF7 are upregulated in *ddc* but not in *met1*. Similarly, HY5 and HYH levels are reduced in both mutants, indicating a dampening of early photomorphogenic signalling [24]. Non-CG methylation loss particularly affects auxin- and light-related transcription, while some core circadian regulators are downregulated in both *met1* and *ddc*, suggesting a link between epigenetic control and temporal regulation of growth [25,26]. These observations point to DNA methylation as a potential integrator of light, hormone, and circadian signalling pathways.

Environmental factors further influence methylation-dependent regulation. Light deficiency has been shown to alter genome-wide methylation patterns, affecting developmental and hormonal gene expression [27]. Stressors such as heavy metals (e.g., Cd) exacerbate hormonal pathway perturbations in wild-type plants [28,29], but also in *ddc* mutants, highlighting the sensitivity of methylation-dependent networks to environmental cues [22,30]. Together, these studies suggest that DNA methylation may act as a molecular interface between environmental perception and growth regulation [31]. Despite accumulating evidence linking DNA methylation to hormone and light signalling, a comprehensive analysis integrating photomorphogenic responses, hormone sensitivity, and transcriptional regulation during early seedling development is still lacking. However, a systematic comparison of CG versus non-CG DNA methylation mutants integrating photomorphogenic responses, hormone sensitivity, and transcriptional regulation during early seedling development has not yet been performed.

This study investigated the influence of disruptions to CG and non-CG DNA methylation on auxin- and GA-dependent seedling development, light-mediated morphogenesis, and circadian-related transcriptional networks in *A. thaliana*. *ddc* mutants displayed shorter hypocotyls, and organ-specific defects in cotyledon expansion. Exogenous auxin and GA treatments revealed subtle differences in sensitivity between mutants and wild-type, while quantitative real-time PCR (qRT-PCR) profiling indicated widespread misregulation of auxin- and light-related genes, with *ddc* showing broader transcriptional perturbations. By directly comparing CG and non-CG methylation mutants under controlled light and hormone conditions, our results provide new insight into how epigenetic regulation fine-tunes developmental plasticity.

Taken together, these findings suggest a mechanistic link between DNA methylation, hormonal crosstalk, and environmental responsiveness, highlighting the importance of epigenetic regulation in the precise control of plant development and phenotypic plasticity. More broadly, this work contributes to a better understanding of how epigenetic mechanisms shape the integration of environmental and endogenous signals during early development, a process that is central to plant adaptability and growth robustness under fluctuating conditions.

## 2. Results

### 2.1. Hypocotyl and Cotyledon Responses Under Continuous Light

**Figure 1:**
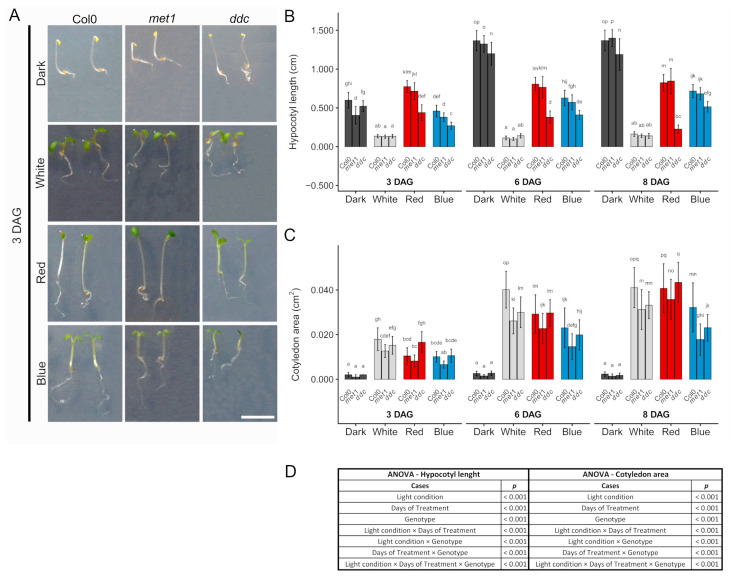
Light-dependent growth regulation of seedling growth in Col0, *met1* and *ddc*. (**A**) Representative images of *A. thaliana* seedlings grown for 3 days after germination (3 DAG) under dark, white, red, or blue light conditions. Seedlings of the wild-type (Col0) and the DNA methylation mutants *met1* and *ddc* are shown. Scale bar: 0.5 cm. (**B**) Quantification of hypocotyl length and (**C**) cotyledon area of seedlings grown under the indicated light conditions at 3, 6, and 8 days after germination (DAG). Bars represent mean ± SD. (**D**) Statistical significance was assessed using three-way analysis of variance (ANOVA) with Light condition, Days of Treatment, and Genotype as fixed factors, including their interaction (Light condition × Days of Treatment × Genotype). When significant effects were detected, post hoc comparisons were performed using Tukey’s multiple comparison test. Different letters indicate statistically significant differences (*p* < 0.05).

To evaluate the effect of light quality on early seedling development and to determine whether defects in DNA methylation alter these responses, seedlings of *A. thaliana* (Col0, *met1*, and *ddc*) were grown for 3, 6, and 8 days after germination (DAG) under white, red, blue, or dark conditions. This experimental framework allowed us to assess how light-driven developmental programmes interact with methylation-dependent regulation. Clear light-dependent patterns of hypocotyl elongation were observed in seedlings across all genotypes and time points (Figure 1). This growth reflected classical photomorphogenic responses: initially, the shortest hypocotyls were observed under continuous white light; intermediate elongation occurred under blue light and in darkness; and maximal elongation occurred under red light (Figure 1A,B). However, at 6 and 8 days after germination, seedlings grown in darkness showed the longest hypocotyls compared with the other conditions, whereas under white light conditions, hypocotyls were always the shortest. These trends were consistently observed in Col0, *met1*, and *ddc* seedlings at 3, 6, and 8 days after germination. Genotypic differences were detectable across most light conditions. Col0 generally exhibited the longest hypocotyls, whereas *ddc* seedlings showed a pronounced reduction in elongation under red and blue light conditions. The *met1* mutant displayed an intermediate phenotype, with hypocotyl lengths not severely reduced as in *ddc*. Differences among the genotypes became more evident over time, particularly at 6 and 8 days after germination (Figure 1B). Under continuous white light, hypocotyl elongation was strongly suppressed in all genotypes, and genotypic differences were not significant. In contrast, under red and blue light, *ddc* showed significantly reduced hypocotyl elongation, indicating an altered responsiveness to specific light qualities. In darkness, although all genotypes exhibited strong elongation, *ddc* seedlings remained significantly shorter than Col0, suggesting a reduced capacity for skotomorphogenic growth (Figure 1B).

Cotyledon expansion also showed differences related to light conditions and genotype, with the largest cotyledons observed under continuous white light, slightly smaller cotyledons under red light, and further reduced expansion under blue light, whereas dark-grown seedlings developed uniformly small cotyledons (Figure 1C). These trends were consistently observed in Col0, *met1*, and *ddc* seedlings at 3, 6, and 8 DAG. Genotypic differences were detectable but limited, as both *met1* and *ddc* displayed reduced cotyledon areas relative to Col0, although not always significant. The *ddc* mutant displayed an intermediate phenotype, whereas *met1* showed a more severe phenotype. Genotypic differences became more pronounced over time, particularly at 6 and 8 DAG. Under continuous white light, cotyledon expansion was maximal in all genotypes, whereas blue light supported reduced expansion. Red light induced intermediate cotyledon growth. In darkness, cotyledon expansion was almost completely suppressed at all time points. Unlike hypocotyl elongation, cotyledon expansion continued between 6 and 8 DAG under all light conditions, indicating sustained photomorphogenic growth (Figure 1C). Overall, these results suggest that light quality influences hypocotyl elongation and cotyledon expansion, with darkness promoting hypocotyl elongation and limiting cotyledon growth. Differences between Col0, *met1*, and *ddc* were frequently observed for both traits, suggesting that altered DNA methylation can modulate early light-dependent seedling development.

Significant light condition × days of treatment × genotype interactions for both hypocotyl length and cotyledon area indicate that DNA methylation contributes to the temporal modulation of light-dependent growth responses in an organ-specific manner during early seedling development.

### 2.2. Growth Responses to Exogenous Auxin (IAA)

**Figure 2:**
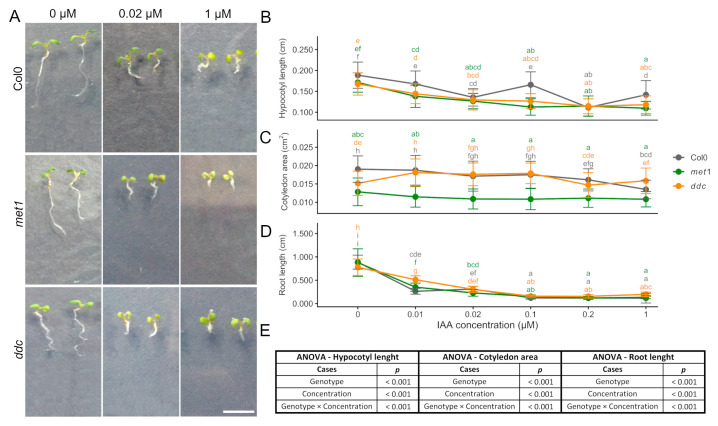
Auxin dose–dependent regulation of seedling growth in Col0, *met1*, and *ddc*. (**A**) Representative images of *A. thaliana* seedlings grown for 6 days after germination (6 DAG) in the presence of increasing concentrations of indole-3-acetic acid (IAA) under long-day conditions. Seedlings of the wild-type (Col0) and the DNA methylation mutants *met1* and *ddc* are shown. Scale bar: 0.5 cm. (**B**) Hypocotyl length, (**C**) cotyledon area, and (**D**) root length of *A. thaliana* seedlings grown in the presence of increasing concentrations of IAA. Measurements were performed at 6 DAG. Data are presented as mean ± SD. (**E**) Statistical significance was assessed using two-way analysis of variance (ANOVA) with Genotype and IAA concentration as fixed factors, including their interaction (Genotype × Concentration). When significant effects were detected, post hoc comparisons were performed using Tukey’s multiple comparison test. Different letters indicate statistically significant differences (*p* < 0.05).

To evaluate the effect of exogenous auxin on early seedling development, hypocotyl length, cotyledon area, and primary root length were analysed in Col0, *met1*, and *ddc* seedlings grown in the presence of increasing concentrations of indole-3-acetic acid (IAA) (Figure 2). Across all genotypes, IAA treatment resulted in a general inhibition of growth in all three organs at concentrations > 0.01 µM, confirming the well-established inhibitory effect of exogenous auxin at higher concentrations. Two-way ANOVA revealed that auxin concentration exerted a highly significant effect on hypocotyl length, cotyledon area, and root length, indicating that growth responses were strongly dose-dependent (Figure 2E).

For hypocotyl length (Figure 2A,B), both genotype and concentration had significant effects, and a significant genotype × concentration interaction was detected. Hypocotyl elongation decreased progressively with increasing IAA concentration across all genotypes; however, differences between Col0 and the methylation mutants were most pronounced at low to intermediate auxin doses and became less pronounced at higher concentrations, at which growth inhibition converged among genotypes. This interaction indicates that the magnitude of auxin-mediated inhibition differed across genotypes with respect to the applied concentration.

In the cotyledon area (Figure 2A,C), two-way ANOVA revealed significant effects of genotype, concentration, and genotype × concentration interaction. Although the cotyledon area tended to decrease with increasing IAA concentration, the response was relatively mild and variable. *met1* seedlings consistently displayed smaller cotyledons than Col0 and *ddc* across concentrations, indicating a genotype-dependent baseline difference rather than a dose–response difference.

Primary root growth was the most sensitive trait to auxin treatment (Figure 2A,D). Two-way ANOVA indicated a highly significant effect of auxin concentration, as well as a significant genotype effect and genotype × concentration interaction. Root elongation was strongly inhibited at higher IAA concentrations across all genotypes. Although some variability was observed at intermediate doses, genotypic differences diminished as auxin levels increased, resulting in comparable inhibition at the highest concentrations tested.

While exogenous auxin treatments revealed genotype-dependent differences in growth inhibition, hormone-mediated regulation of seedling development relies on both hormone biosynthesis and transport as well as on crosstalk with other growth-promoting pathways. We therefore next examined gibberellin-mediated growth responses, followed by an analysis of the effects of polar auxin transport inhibition.

These results demonstrate that increasing IAA concentrations inhibit hypocotyl elongation, cotyledon expansion, and primary root growth in all genotypes. While auxin concentration represents the dominant factor controlling growth inhibition, genotype-dependent differences are evident, particularly for hypocotyl elongation at low to intermediate IAA levels. The presence of a significant genotype × concentration suggests that DNA methylation status partially modulates auxin sensitivity in an organ-specific manner during early seedling development.

### 2.3. Growth Responses to Exogenous Gibberellin (GA_3_)

**Figure 3:**
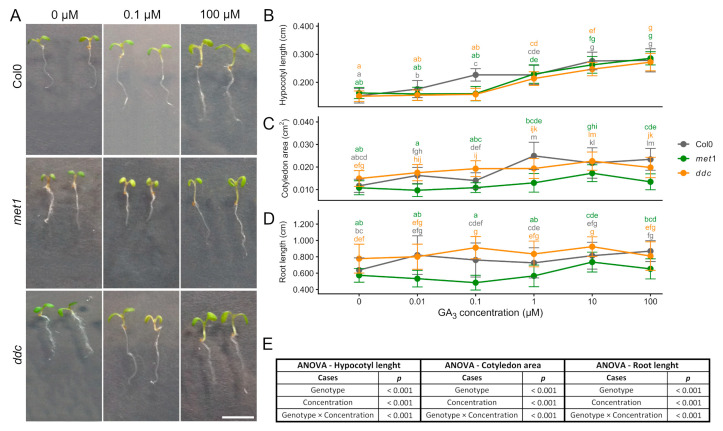
Gibberellin dose–dependent regulation of seedling growth in Col0, *met1,* and *ddc*. (**A**) Representative images of *A. thaliana* seedlings grown for 6 days after germination (6 DAG) in the presence of increasing concentrations of gibberellic acid (GA_3_) under long-day conditions. Seedlings of the wild-type (Col0) and the DNA methylation mutants *met1* and *ddc* are shown. Scale bar: 0.5 cm. (**B**) Hypocotyl length, (**C**) cotyledon area, and (**D**) root length of *A. thaliana* seedlings grown in the presence of increasing concentrations of GA_3_. Measurements were performed at 6 DAG. Data are presented as mean ± SD. (**E**) Statistical significance was assessed using two-way analysis of variance (ANOVA) with Genotype and GA_3_ concentration as fixed factors, including their interaction (Genotype × Concentration). When significant effects were detected, post hoc comparisons were performed using Tukey’s multiple comparison test. Different letters indicate statistically significant differences (*p* < 0.05).

To assess how disruptions in DNA methylation affect gibberellin-mediated growth, we quantified hypocotyl, cotyledon, and root responses of *A. thaliana* seedlings (Col0, *met1*, and *ddc*) to increasing concentrations of GA_3_ (Figure 3).

Two-way ANOVA revealed that GA_3_ concentration significantly affected hypocotyl length, cotyledon area, and root length, confirming a strong dose-dependent effect of gibberellin on seedling growth across all genotypes (Figure 3E).

Hypocotyl length generally increased with GA_3_ concentration in all genotypes. Growth stimulation became more evident at intermediate to high concentrations, with *ddc* and *met1* showing a similar overall trend. Initially, *met1* and *ddc* hypocotyls exhibited a weaker response at low concentrations, followed by increased elongation at higher doses, resulting in partial convergence with the wild-type (Figure 3A,B). Statistical analysis indicated that hypocotyl elongation was significantly influenced by GA_3_ concentration, and a significant genotype × concentration interaction was detected, indicating that the magnitude of the elongation response differed among genotypes depending on the applied GA_3_ concentration (Figure 3B,E).

Cotyledon area showed a more variable response to GA_3_. In Col0, cotyledon expansion increased at intermediate concentrations and then stabilised. *met1* and *ddc* showed increases at low to intermediate doses and no further enhancement at the highest concentration tested (Figure 3A,C). Two-way ANOVA revealed significant effects of both genotype and GA_3_ concentration on cotyledon area, and the genotype × concentration interaction was also significant, indicating that GA_3_-dependent cotyledon expansion followed a broadly different dose–response pattern across genotypes, accordingly with the persistent baseline differences among lines (Figure 3C,E).

Primary root length also responded to GA_3_, although the pattern differed among genotypes. Among the genotypes analysed, *ddc* displayed the most pronounced responsiveness to GA_3_, whereas Col0 showed an intermediate response, with a modest increase in root length at higher GA_3_ concentrations (Figure 3A,D). Notably, *met1* showed no appreciable increase in root length at low GA_3_ concentrations, in contrast to the WT, which already exhibited a clear elongation response under the same conditions, indicating a reduced sensitivity to the hormone in *met1*. A partial recovery of root elongation became evident only at 10 µM, rather than at the highest concentration tested (Figure 3D). Accordingly, root length was significantly affected by GA_3_ concentration and genotype, and a significant genotype × concentration interaction was detected, indicating genotype-specific differences in the dose-dependent regulation of root elongation (Figure 3D,E).

Overall, GA_3_ promotes elongation in hypocotyls and roots and expansion in cotyledons in all genotypes, with non-CG methylation loss in *ddc* modulating the dose-dependent response in an organ-specific manner. In particular, the presence of significant genotype × concentration interactions for hypocotyl, cotyledon, and root growth supports the conclusion that DNA methylation status influences the sensitivity and amplitude of gibberellin-mediated growth responses in a tissue-dependent manner.

### 2.4. Growth Responses to the Auxin Transport Inhibitor TIBA

**Figure 4:**
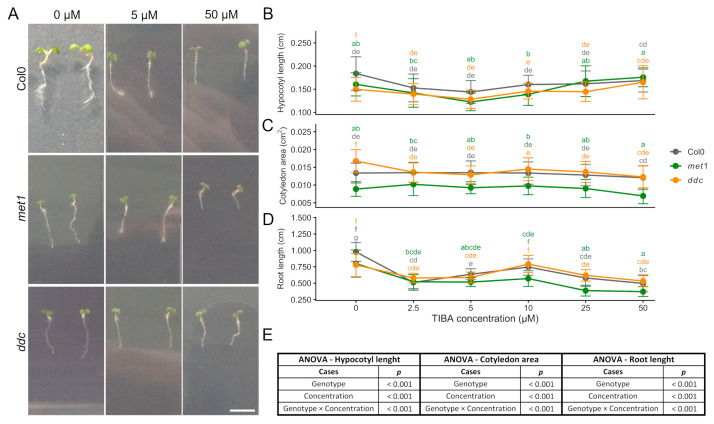
TIBA dose–dependent regulation of seedling growth in Col0, *met1,* and *ddc*. (**A**) Representative images of *A. thaliana* seedlings grown for 4 days after germination (DAG) in control medium and then transferred for 2 days to medium enriched with increasing concentrations of 2,3,5-triiodobenzoic acid (TIBA) under long-day conditions. Seedlings of the wild-type (Col0) and the DNA methylation mutants *met1* and *ddc* are shown. Scale bar: 0.5 cm. (**B**) Hypocotyl length, (**C**) cotyledon area, and (**D**) root length of *A. thaliana* seedlings grown in the presence of increasing concentrations of TIBA. Measurements were performed at 6 DAG. Data are presented as mean ± SD. (**E**) Statistical significance was assessed using two-way analysis of variance (ANOVA) with Genotype and TIBA concentration as fixed factors, including their interaction (Genotype × Concentration). When significant effects were detected, post hoc comparisons were performed using Tukey’s multiple comparison test. Different letters indicate statistically significant differences (*p* < 0.05).

To evaluate the effect of polar auxin transport inhibition on early seedling development, *A. thaliana* seedlings (Col0, *met1*, and *ddc*) were exposed to increasing concentrations of 2,3,5-triiodobenzoic acid (TIBA) (0–50 μM), and hypocotyl, cotyledon, and root growth were quantified (Figure 4). Two-way ANOVA revealed that TIBA concentration significantly affected hypocotyl length, cotyledon area, and root length, indicating a strong dose-dependent impact of auxin transport inhibition on seedling growth across all genotypes (Figure 4E). Hypocotyl elongation decreased progressively up to 5 μM TIBA in all genotypes. However, at higher concentrations, *met1* and *ddc* hypocotyls elongated similarly to WT, reaching lengths comparable to Col0 at 50 μM for *ddc*, and exceeding it for *met1*. Except for the highest concentrations, both mutants were generally shorter than WT, highlighting genotype-specific compensatory growth responses (Figure 4A,B). Statistical analysis indicated that hypocotyl length was significantly influenced by both genotype and TIBA concentration, and a significant genotype × concentration interaction was detected, indicating that the extent of hypocotyl growth inhibition and recovery differed among genotypes depending on TIBA concentration (Figure 4B,E).

Cotyledon area showed a relatively limited response to TIBA. In Col0 and *ddc*, cotyledon size remained relatively stable across the concentration range. In *met1*, cotyledon area varied modestly with concentration. It was consistently lower compared to the other ones (Figure 4A,C). Two-way ANOVA revealed a significant effect of genotype on cotyledon area, TIBA concentration, and the genotype × concentration interaction, indicating that cotyledon growth differences reflect both genotype-dependent baseline variation and differential sensitivity to auxin transport inhibition (Figure 4C,E).

Primary root growth was strongly inhibited by TIBA in all genotypes. WT and *ddc* roots showed a biphasic response, with initial reduction at low concentrations, slight recovery at intermediate doses, and subsequent decline at high concentrations. In contrast, *met1* roots remained consistently shorter across the entire concentration range. Although a slight increase in root length was observed at 10 µM TIBA, this change was not statistically significant, and root growth remained strongly inhibited at higher concentrations (Figure 4A,D). Accordingly, root length was significantly affected by TIBA concentration and genotype, and by genotype × concentration interaction, indicating a broadly conserved dose-dependent inhibition of root elongation across genotypes (Figure 4D,E).

Although exogenous auxin and inhibition of polar auxin transport are often expected to produce opposite outcomes, comparison of the IAA and TIBA treatments indicates that this opposition is only partial: both treatments similarly inhibit root growth, whereas hypocotyl responses diverge at higher concentrations, consistent with TIBA affecting auxin distribution rather than simply reducing auxin levels (Figure 2 and Figure 4). No significant differences were observed between mock-treated samples across all experiments, indicating that the solvents alone did not affect the measured phenotypes (Supplementary Figure S1).

Overall, TIBA treatment affected hypocotyl, cotyledon, and root growth in a concentration- and organ-dependent manner. While differences between Col0, *met1*, and *ddc* were observed under specific conditions, responses to auxin transport inhibition were broadly similar among genotypes at higher TIBA concentrations. In particular, the presence of a significant genotype × concentration interaction supports the conclusion that DNA methylation status modulates compensatory growth responses to disrupted auxin transport in an organ-specific manner.

### 2.5. Gene Expression Profiling Reveals Distinct Light, Hormone, and Clock Signalling Alterations in DNA Methylation Mutants

**Figure 5:**
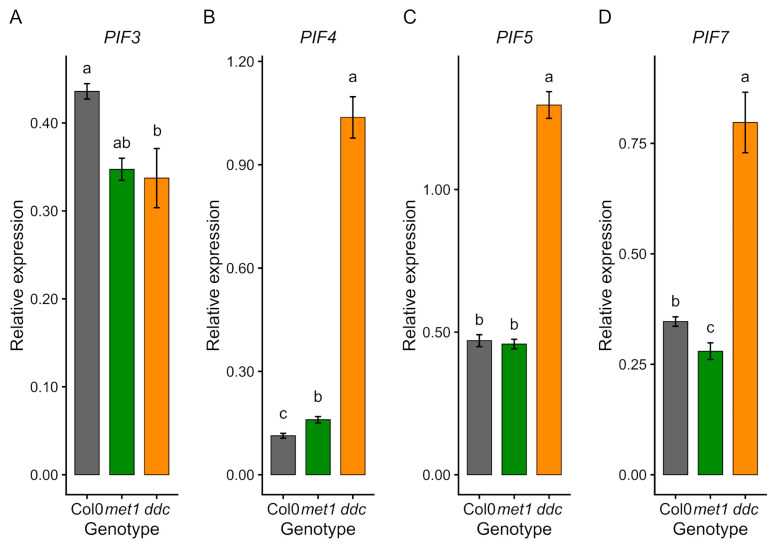
qRT–PCR validation of Phytochrome-Interacting Factor (PIF) gene expression in Col0, *met1,* and *ddc*. Relative expression levels of *PIF3* (**A**), *PIF4* (**B**), *PIF5* (**C**), and *PIF7* (**D**) were measured by quantitative RT–PCR in wild-type (Col0) and DNA methylation mutants *met1* and *ddc*. Expression levels (2^−ΔCt^) are shown as mean ± SE of three independent biological replicates. Statistical analyses were performed by one-way ANOVA followed by Tukey’s post hoc test (*p* < 0.05) on ΔCt values. Different lowercase letters indicate statistically significant differences among genotypes.

**Figure 6:**
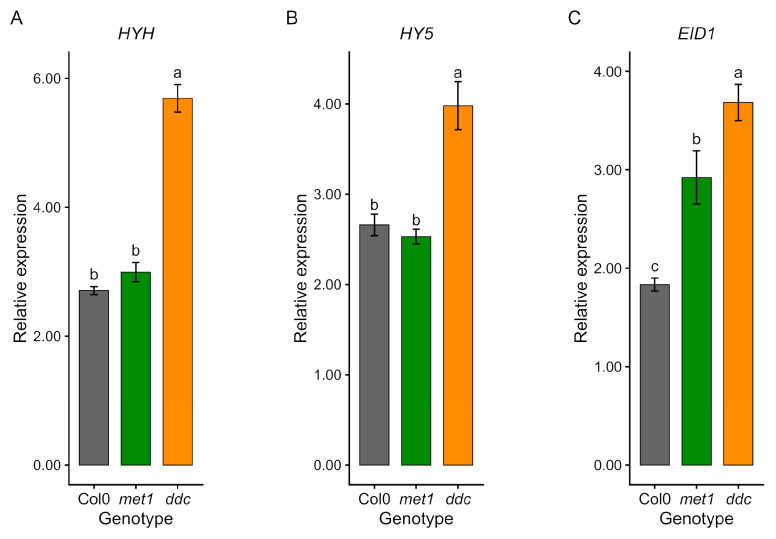
qRT–PCR validation of light-signalling-related gene expression in Col0, *met1,* and *ddc*. Relative expression levels of *HYH* (**A**), *HY5* (**B**), and *EID1* (**C**) were measured by quantitative RT–PCR in wild-type (Col0) and DNA methylation mutants *met1* and *ddc*. Expression levels (2^−ΔCt^) are shown as mean ± SE of three independent biological replicates. Statistical analyses were performed by one-way ANOVA followed by Tukey’s post hoc test (*p* < 0.05) on ΔCt values. Different lowercase letters indicate statistically significant differences among genotypes.

**Figure 7:**
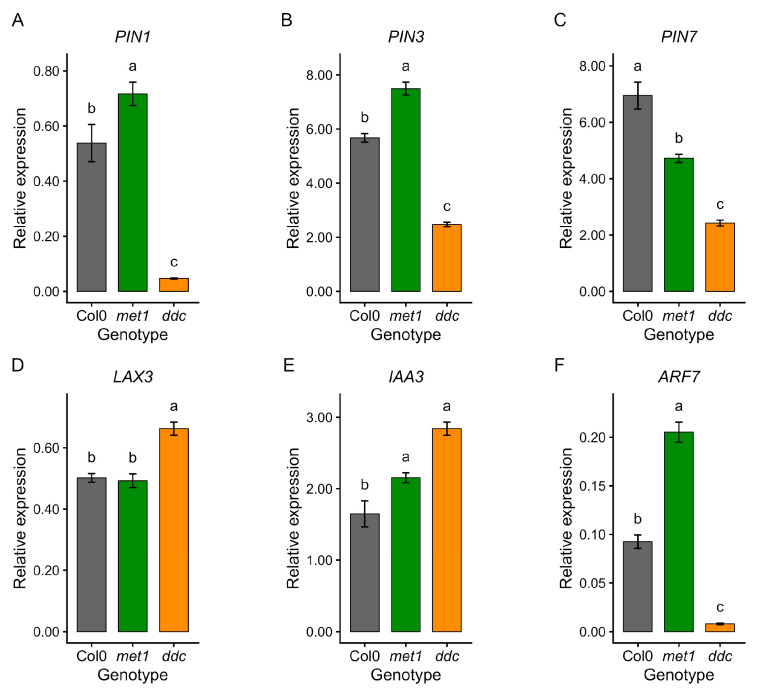
qRT–PCR validation of auxin-related gene expression in Col0, *met1,* and *ddc*. Relative expression levels of *PIN1* (**A**), *PIN3* (**B**), *PIN7* (**C**), *LAX3* (**D**), *IAA3* (**E**), and *ARF7* (**F**) were measured by quantitative RT–PCR in wild-type (Col0) and DNA methylation mutants *met1* and *ddc*. Expression levels (2^−ΔCt^) are shown as mean ± SE of three independent biological replicates. Statistical analyses were performed by one-way ANOVA followed by Tukey’s post hoc test (*p* < 0.05) on ΔCt values. Different lowercase letters indicate statistically significant differences among genotypes.

**Figure 8:**
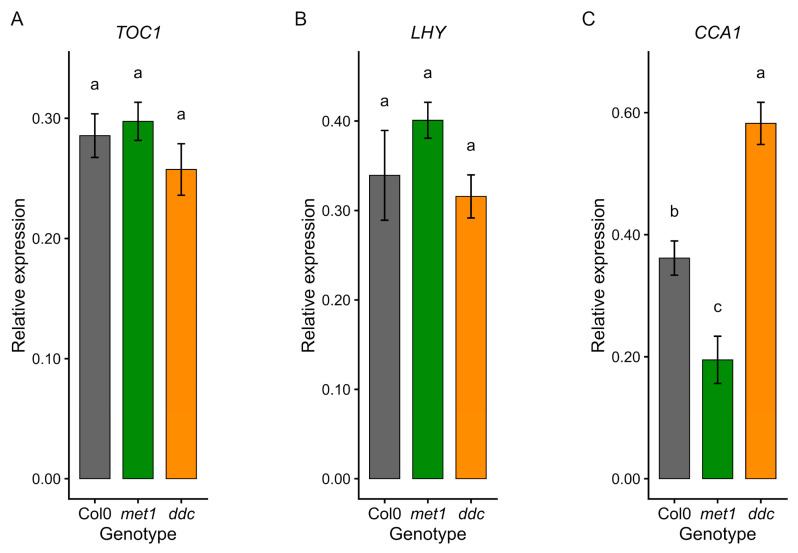
qRT–PCR analysis of circadian clock gene expression in Col0, *met1,* and *ddc*. Relative expression levels of *TOC1* (**A**), *LHY* (**B**), and *CCA1* (**C**) were measured by quantitative RT–PCR in wild-type (Col0) and DNA methylation mutants *met1* and *ddc*. Expression levels (2^−ΔCt^) are shown as mean ± SE of three independent biological replicates. Statistical analyses were performed by one-way ANOVA followed by Tukey’s post hoc test (*p* < 0.05) on ΔCt values. Different lowercase letters indicate statistically significant differences among genotypes.

To link the morphological and hormonal responses to underlying molecular changes, we quantified the expression of genes involved in light perception, auxin signalling, and circadian regulation in Col0, *met1,* and *ddc* (Figure 5, Figure 6, Figure 7 and Figure 8).

Analysis of Phytochrome-interacting factors (PIFs) revealed distinct and genotype-specific transcriptional patterns (Figure 5). *PIF3* expression was reduced in both mutants compared with Col0 (Figure 5A). In contrast, *PIF4*, *PIF5*, and *PIF7* showed divergent regulation between mutants: *ddc* displayed markedly increased transcript levels of all three genes (Figure 5B–D), whereas *met1* showed reduced expression, comparable with wild-type. These opposing trends are consistent with distinct regulatory outcomes associated with preferential loss of CG methylation in *met1* and non-CG methylation in *ddc*.

Genes involved in early light signalling also exhibited altered expression (Figure 6). *HYH* and *HY5* expression differed between genotypes, with elevated transcript levels in *ddc* and values close to Col0 in *met1* (Figure 6A,B). *EID1* transcripts were increased in both mutants, particularly in *ddc* (Figure 6C). Together, these data indicate that disruption of DNA methylation, particularly non-CG methylation, affects multiple components of photomorphogenic signalling rather than simply attenuating light responses.

Auxin-related genes showed the most pronounced differences between genotypes (Figure 7). In *ddc*, the genes encoding auxin efflux carriers *PIN1*, *PIN3*, and *PIN7* were strongly downregulated comparable to Col0, whereas *met1* showed either moderate reductions (*PIN7*) or increased expression (*PIN1* and *PIN3*) (Figure 7A–C). In contrast, the auxin influx carrier *LAX3* and the early auxin-responsive gene *IAA3* were upregulated in *ddc*, while showing smaller or no changes in *met1* (Figure 7D,E). Instead, *ARF7* expression was markedly reduced in *ddc* but significantly increased in *met1* (Figure 7F), highlighting opposite regulatory outcomes for this key auxin response factor. These transcriptional differences are consistent with the distinct auxin sensitivities observed in growth assays.

Finally, expression of core circadian clock components showed gene- and genotype-specific effects (Figure 8). *TOC1* and *LHY* transcript levels were globally similar to Col0 in both mutants (Figure 8A,B), whereas *CCA1* expression was reduced in *met1* but increased in *ddc* (Figure 8C). This suggests that DNA methylation perturbations impact individual clock components rather than uniformly suppressing circadian gene expression.

Overall, these results indicate that loss of CG and non-CG DNA methylation leads to distinct transcriptional reprogramming across light, auxin, and circadian pathways. While both *met1* and *ddc* show alterations in light- and clock-associated genes, *ddc* exhibits a broader and more pronounced disruption of auxin-related transcription, consistent with its stronger and more variable hormonal and developmental phenotypes.

## 3. Discussion

### 3.1. DNA Methylation as a Quantitative Regulator of Early Seedling Development

This study demonstrates that DNA methylation functions as a quantitative fine-tuner of early *A. thaliana* seedling development, modulating the amplitude, sensitivity, and temporal dynamics of growth responses to light, hormonal cues, and circadian regulation. By integrating morphological, hormonal, light-dependent, and transcriptional analyses and directly contrasting *met1* mutants impaired in CG methylation maintenance with *ddc* mutants lacking non-CG methylation through combined loss of *DRM1*, *DRM2*, and *CMT3*, we show that CG and non-CG methylation layers play distinct yet partially overlapping roles in shaping organ- and context-dependent developmental plasticity during the transition from germination to photomorphogenesis [32]. Importantly, the phenotypic effects observed in both CG and non-CG methylation mutants do not reflect a complete disruption of developmental programmes, but rather quantitative changes in response amplitude, sensitivity, and organ specificity, supporting a modulatory role of DNA methylation in early development.

Together, these results establish robust phenotypic and transcriptional associations, while the mechanistic interpretations developed below should be viewed as integrative models rather than direct causal relationships.

### 3.2. CG and Non-CG Methylation Effects on Photomorphogenic Growth

Defects in both CG and non-CG methylation altered classical photomorphogenic traits in a manner dependent on light quality. At 6 DAG, the overall hierarchy of hypocotyl elongation, shortest in white light and longest in darkness, was conserved across white, red, blue, and dark conditions, indicating that the core architecture of light perception remains intact in methylation mutants. Accordingly, *met1* and *ddc* retained fundamental light responses, consistent with the robustness of core photomorphogenic signalling pathways to epigenetic perturbation [23,24,33]. Nevertheless, subtle but progressive differences in hypocotyl and cotyledon growth, particularly under low-light conditions, revealed genotype-specific modulation of developmental plasticity. These differences became more pronounced over time, suggesting that DNA methylation primarily affects cumulative growth outcomes rather than immediate developmental decisions [34]. Similar phenomena have been reported in sunflower seedlings, where rapid photoperiod entrainment occurs after minimal light exposure, likely exploiting pre-existing basic circadian rhythms [35,36,37,38].

### 3.3. DNA Methylation Fine-Tunes Light-Quality–Dependent Growth Amplitude

Both *met1* and *ddc* seedlings consistently exhibited reduced hypocotyl elongation, particularly under red and blue light, with the strongest reduction observed in *ddc.* This indicates that DNA methylation fine-tunes the amplitude of growth responses downstream of light perception rather than acting as a binary switch. Red and blue light act via phytochromes and cryptochromes on transcriptional regulators such as PIFs, HY5, and HYH [39,40,41,42], and the stronger impairment in *ddc* is consistent with the misregulation of PIF family members observed in this mutant. In contrast, *met1* displayed intermediate phenotypes, suggesting partial functional redundancy or compensatory mechanisms when CG methylation alone is compromised. Genotypic differences in hypocotyl length were smaller under continuous white light, likely because simultaneous activation of multiple photoreceptors buffers defects in individual signalling branches. This supports the idea that light deficiency can alter methylation patterns, influencing developmental responses to environmental cues [27]. Cotyledon expansion followed a different pattern: although both mutants displayed reduced cotyledon area compared with Col0, *ddc* did not consistently show stronger defects as in *met1,* highlighting organ-specific sensitivity to epigenetic perturbations. Given that hypocotyl elongation primarily depends on cell elongation, whereas cotyledon expansion involves both cell division and differentiation [43,44], these processes may rely on distinct methylation-sensitive transcriptional programmes.

### 3.4. Hormonal and Transcriptional Regulation by CG and Non-CG DNA Methylation

Auxin treatments revealed that DNA methylation status significantly influences organ-specific auxin sensitivity. Exogenous IAA inhibited growth in all genotypes, confirming auxin concentration as the dominant determinant of growth inhibition. However, genotype-dependent effects emerged at low to intermediate doses, indicating that DNA methylation modulates auxin sensitivity thresholds rather than maximal responsiveness. Significant genotype × concentration interactions, particularly in hypocotyls, showed stronger inhibition in *met1* and *ddc* at low IAA doses, with convergence at higher concentrations.

Transcriptional data support this interpretation. In *ddc*, auxin efflux carriers (*PIN1*, *PIN3*, *PIN7*) were downregulated, while *LAX3* and *IAA3* were upregulated, consistent with disrupted auxin transport and compensatory feedback [45,46,47]. These changes are consistent with altered intracellular auxin distribution and align with reports that non-CG methylation is critical for proper auxin distribution and signalling. This affects the transcription of PIN efflux carriers and ARFs [20,21,48], as well as prior observations that epigenetic modifications fine-tune hormone signalling outputs without abolishing pathway function [32,49]. ARF transcription factors, including ARF6, ARF7/NPH4, and ARF8, play a key role in auxin-dependent hypocotyl elongation by interacting with *SAUR* genes, cell wall-loosening enzymes and components of GA and brassinosteroid pathways [50,51]. They also play a role under conditions that induce rapid growth, such as darkness, shade, or elevated temperature [52,53,54,55]. Altered auxin sensitivity in *ddc* may reflect modulation of ARF-mediated transcriptional networks by non-CG DNA methylation. Root responses were less genotype-dependent, likely due to the inherently high auxin sensitivity of this organ.

Gibberellin treatments further highlighted the differential contributions of CG and non-CG methylation to hormone responsiveness. GA_3_ promoted elongation in hypocotyls and roots and stimulated cotyledon expansion across genotypes, but with distinct dose-dependent responses. Significant genotype × concentration interactions indicate that DNA methylation modulates GA_3_ responsiveness in a tissue-specific manner. In hypocotyls, *met1* and *ddc* required higher GA_3_ concentrations to achieve elongation comparable to Col0, suggesting reduced sensitivity rather than impaired signalling. Root growth responses to GA_3_ differed markedly: *ddc* showed enhanced elongation, whereas *met1* exhibited reduced responsiveness at higher GA_3_ doses, indicating a prominent role for non-CG methylation in maintaining appropriate GA_3_ sensitivity in roots. The balance between elongation and differentiation relies heavily on finely tuned hormone gradients and transcriptional regulation [18,56]. The altered methylation status in *ddc* may therefore disrupt this balance. Cotyledon expansion was comparatively robust, supporting the notion that this process relies less on precise hormonal thresholds.

Treatment with the auxin transport inhibitor TIBA revealed additional layers of epigenetically modulated growth plasticity. While root growth was strongly inhibited across all genotypes, hypocotyl responses were genotype-dependent. At high TIBA concentrations, *met1* and *ddc* partially recovered elongation, with met1 exceeding Col0, indicating activation of alternative growth pathways. These findings suggest that DNA methylation contributes to feedback regulation and adaptive plasticity under perturbed hormone distribution [57,58], although the underlying molecular mechanisms remain inferential. Cotyledon responses were largely genotype-independent, reinforcing organ-specific epigenetic regulation.

Gene expression analyses provided a unifying framework for interpreting the phenotypic differences. *ddc* showed strong upregulation of *PIF4*, *PIF5*, and *PIF7*, coupled with downregulation of *PIF3*, whereas *met1* displayed milder changes. Opposite regulation of *ARF7* in *ddc* and *met1* further underscores the distinct transcriptional consequences of CG versus non-CG methylation [59,60,61,62,63,64]. Clock gene expression was selectively affected: *TOC1* and *LHY* remained stable, while *CCA1* showed opposite trends in the two mutants, suggesting modulation of specific clock components rather than global circadian disruption [25,26,65,66]. As expression was assessed at a single time point, changes in phase or amplitude cannot be excluded, but the gene-specific nature of the effects argues against collapse of circadian regulation [36,67].

## 4. Materials and Methods

### 4.1. Plant Material and Growth Conditions

Seeds of *A. thaliana* ecotype Columbia (Col0) and the DNA methylation mutants *met1* and *drm1*, *drm2*, and *cmt3* (*ddc*) were used in this study. Seeds of the *met1* and *ddc* mutants were purchased from the Nottingham *Arabidopsis* Stock Centre (NASC) (Loughborough, UK). Seedlings were grown on solid medium containing half-strength Murashige and Skoog (MS) basal salts with Gamborg’s vitamins (Sigma-Aldrich, St. Louis, MO, USA), supplemented with 0.1 g L^−1^ myo-inositol, 0.5 g L^−1^ MES, and 1% (*w*/*v*) sucrose, and 0.85% agar (*w*/*v*) (pH 5.7). Seeds were surface sterilised by incubation in 50% (*v*/*v*) ethanol for 3 min, followed by 0.01% (*v*/*v*) sodium hypochlorite, and rinsed five times with sterile distilled water, then individually pipetted onto solid medium under sterile conditions. Preliminary experiments on hypocotyl length and cotyledon area were conducted as described in Woloszynska et al. (2018) [68]. Seeds were first exposed to white light (100 µmol m^−2^ s^−1^) for 6 h to induce germination and were then transferred to continuous dark or continuous white, red, or blue light (all at 100 µmol m^−2^ s^−1^). White light was provided by cool white, fluorescent lamps (Philips, Amsterdam, The Netherlands); red light was obtained by filtering cool white, fluorescent light through red plastic (Rohm and Haas, Philadelphia, PA, USA) and red cellophane (UCB-Sidac, Gent, Belgium); and blue light was supplied by dragon tape LEDs (470 nm; Osram, Munich, Germany). Seedlings were grown under these conditions for 3, 6, or 8 days in controlled conditions (20 ± 2 °C, 50% relative humidity). Chemical treatments and relative gene expression analyses were performed on 6-day-old seedlings grown under the same controlled conditions, under long-day photoperiods (16 h light/8 h dark) and continuous light, respectively. For each experiment, three biological replicates were grown for each genotype (Col0, *met1*, and *ddc*), with 30 seedlings per genotype per replicate. Seedlings were randomly assigned to the different treatments. For gene-expression analysis, 6-day-old seedlings were harvested and stored at −80 °C for subsequent gene-expression analysis.

### 4.2. Chemical Treatments

Auxin (indoleacetic-3-acid, IAA) and gibberellin (GA_3_) treatments were performed as previously described by Collett et al. (2000) [69]. Hormone stock solutions (1000×) were prepared in 70% (*v*/*v*) ethanol. IAA was applied at final concentrations of 0, 0.01, 0.02, 0.1, 0.2, and 1 µM, whereas GA_3_ was applied at 0, 0.01, 0.1, 1, 10, and 100 µM. Hormonal treatments were conducted on six-day-old seedlings germinated on enriched medium under long-day conditions. For auxin transport inhibition, the auxin transport inhibitor 2,3,5-triiodobenzoic acid (TIBA) was used; a TIBA stock solution (0.0015 M in 0.1% DMSO) was added to the growth medium to obtain final concentrations of 0, 2.5, 5, 10, 25, and 50 µM. Seedlings were grown for 4 days on standard medium and then transferred to TIBA-containing plates for an additional 2 days prior to phenotypic analysis. Mock control for each experiment was applied by adding the corresponding solvent only (70% ethanol for IAA and GA_3_ treatments, and 0.1% DMSO for TIBA treatments) to the growth medium at the same final concentration used in the hormone-containing media. For each experiment, three biological replicates were grown for each genotype (Col0, *met1*, and *ddc*), with 30 seedlings per genotype per replicate. Seedlings were randomly assigned to the different treatments.

### 4.3. Morphometric Measurements

Seedlings were carefully transferred onto 1% (*w*/*v*) agar plates and scanned using an Epson scanner at a resolution of 600 dpi. Cotyledons were excised, flattened, and scanned separately. Root length, hypocotyl length, and cotyledon area were quantified using ImageJ 1.54p software (https://imagej.net/ij/; accessed on 15 October 2025) as reported in Araniti et al. (2023) [70]. For each experiment, three biological replicates were grown for each genotype (Col0, *met1*, and *ddc*), with 30 seedlings per genotype per replicate. Seedlings were randomly assigned to the different treatments.

### 4.4. RNA Extraction and qRT-PCR Analysis

Total RNA was isolated using the RNeasy Plant Mini Kit (QiAGEN, Hilden, Germany) according to the manufacturer’s instructions. RNA concentration and purity were determined using a NanoDrop spectrophotometer (ND-1000, Thermofisher, Waltham, MA, USA). Complementary DNA (cDNA) was synthesised from 3 µg of total RNA using SuperScript III Reverse Transcriptase (Invitrogen, Milan, Italy). Gene amplification and quantification were performed by qRT-PCR on an Applied Biosystems real-time PCR system in a final volume of 20 µL using Power SYBR^®^ Green PCR Master Mix (Applied Biosystems, Waltham, MA, USA). The housekeeping gene *AT2G28390* (*MONENSIN SENSITIVITY1*, *SAND*) was used as an internal reference for data normalisation [71]. Relative gene expression levels were calculated using the 2^−ΔCt^ method. Primers used are listed in Supplementary Table S1 [21,68,72,73]. For each experiment, three biological replicates were grown for each genotype (Col0, *met1*, and *ddc*), with 30 seedlings per genotype per replicate. Seedlings were randomly assigned to the different treatments.

### 4.5. Statistical Analysis

Each analysis was performed in triplicate, with at least 50 seedlings used for each replicate. Data are presented as the mean ± standard deviation (SD), unless otherwise indicated. Growth parameters under different light conditions were analysed using a three-way analysis of variance (ANOVA) with Light Condition, Days of Treatment, and Genotype as fixed factors, including their interaction. For hormone treatments (IAA, GA_3_, and TIBA), data were analysed using two-way ANOVA with Genotype and Concentration as fixed factors, including their interaction. Post hoc comparisons were performed using Tukey’s test. qRT-PCR data were analysed by one-way ANOVA. For all analyses, differences were considered statistically significant at *p* ≤ 0.05.

## 5. Conclusions

Together, these results indicate that loss of CG methylation mildly attenuates regulatory networks, whereas loss of non-CG methylation leads to broader transcriptional perturbations and enhanced developmental plasticity during early seedling growth [16,20,24]. The stronger phenotypes, altered hormone sensitivities, and compensatory responses observed in *ddc* highlight the importance of RdDM- and CMT-mediated methylation in stabilising gene expression networks integrating environmental and hormonal cues. Differences between CG and non-CG methylation likely reflect their distinct genomic targets, with CG methylation contributing to transcriptional stability within gene bodies and non-CG methylation regulating promoter-proximal regions and transposable elements. Although direct causality between specific methylation changes and individual gene expression outcomes cannot yet be established, the coordinated phenotypic and transcriptional changes strongly support an integrative regulatory role of DNA methylation in early plant development. Future studies combining locus-specific DNA methylation profiling with targeted transcriptional analyses, as well as circadian time-course experiments, will be essential to further resolve the mechanistic and temporal dimensions of this regulation.

## Data Availability

The original contributions presented in this study are included in the article/Supplementary Materials. Further inquiries can be directed to the corresponding author.

## Supplementary Materials

The following supporting information can be downloaded at: https://www.mdpi.com/article/10.3390/ijms27021034/s1.

## Abbreviations

The following abbreviations are used in this manuscript:

ARF	Auxin Response Factor
CCA1	CIRCADIAN CLOCK ASSOCIATED 1
CMT	Chromomethylase
CG	Cytosine–Guanine dinucleotide
CHG/CHH	Non-CG cytosine sequence contexts (H = A, T, or C)
Col0	*Arabidopsis thaliana* Columbia 0 ecotype
DAG	Days After Germination
*ddc*	*drm1–drm2–cmt3* triple mutant
DNMT	DNA Methyltransferase
DRM	Domains Rearranged Methyltransferase
EID1	EMPFINDLICHER IM DUNKELROTEN LICHT 1
GA/GA_3_	Gibberellic Acid
HY5	ELONGATED HYPOCOTYL 5
HYH	HY5 HOMOLOG
IAA	Indole-3-Acetic Acid
LAX	LIKE AUX1 (Auxin Influx Carrier)
LHY	LATE ELONGATED HYPOCOTYL
MET1	Methyltransferase 1
PIF	Phytochrome-Interacting Factor
PIN	PIN-FORMED (Auxin Efflux Carrier)
qRT-PCR	Quantitative Real Time Polymerase Chain Reaction
RdDM	RNA-directed DNA Methylation
TIBA	2,3,5-Triiodobenzoic Acid
TOC1	TIMING OF CAB EXPRESSION
WT	Wild-type
