# Supplementary material for: DNA Methylation Fine-Tunes Light- and Hormone-Responsive Growth Plasticity in Arabidopsis Seedlings
Source: Int J Mol Sci. 2026 Jan 20;27(2):1034. doi: 10.3390/ijms27021034 (PMC12842573; doi:10.3390/ijms27021034)
Supplement: Supplementary file 1 [file ijms-27-01034-s001.zip › Supplementary Figure S1.pdf]

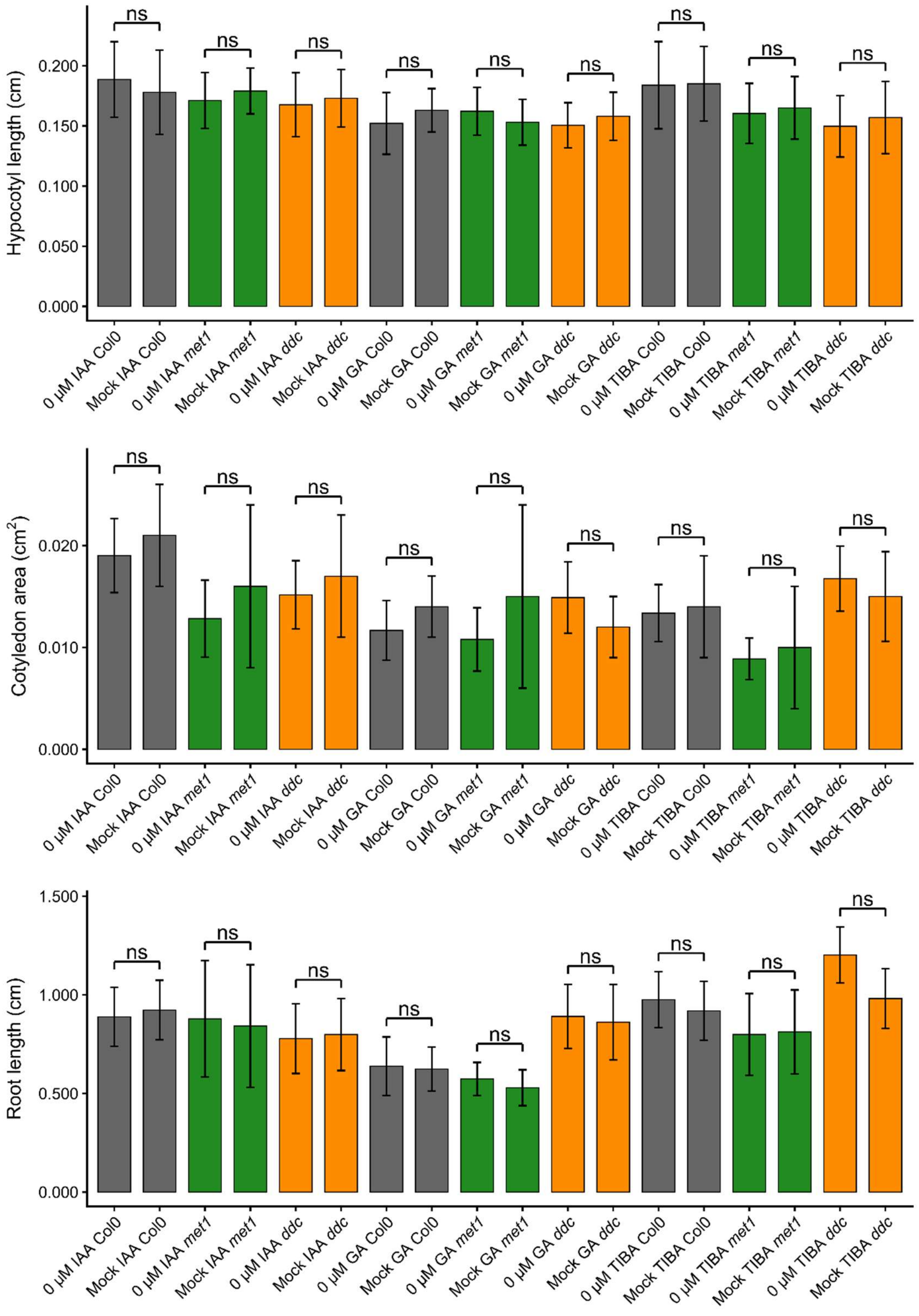

Supplementary Figure S1. Effect of mock treatments on seedling growth in Col0, *met1* and *ddc*. Hypocotyl length (top), cotyledon area (middle) and root length (bottom) were measured in wild-type (Col0) and DNA methylation mutants *met1* and *ddc* grown under mock conditions corresponding to IAA, GA and TIBA treatments. Data are shown as mean ± SD of independent biological replicates. For each genotype, mock-treated seedlings were statistically compared with the corresponding 0 µM control using Student's *t*-test. No statistically significant differences were detected (ns). No hormone was applied in mock conditions.
